# Active topical therapy by “Furuta method” for effective pressure ulcer treatment: a retrospective study

**DOI:** 10.1186/s40780-015-0021-8

**Published:** 2015-07-16

**Authors:** Katsunori Furuta, Fumihiro Mizokami, Hitoshi Sasaki, Masato Yasuhara

**Affiliations:** Department of Clinical Research and Development, National Center for Geriatrics and Gerontology, 7-430 Morioka-cho, Obu, Aichi 474-8511 Japan; Department of Pharmacy, National Center for Geriatrics and Gerontology, Obu, Japan; Department of Hospital Pharmacy, Nagasaki University Hospital, Nagasaki, Japan; Department of Pharmacokinetics and Pharmacodynamics, Graduate School of Medical and Dental Sciences, Tokyo Medical and Dental University, Tokyo, Japan

**Keywords:** Pressure ulcer, Wound physical property, Wound fixation, Furuta method

## Abstract

**Background:**

We newly proposed that “Furuta method,” a pharmacist intervention guidelines, is a topical ointment therapy that considers the physical properties and moist environment of wounds for pressure ulcer (PU) treatment. The aim of this multicenter retrospective study was to investigate the effectiveness of this method for PU.

**Methods:**

A total of 888 consecutive patients who underwent treatment for PU at 37 hospitals and five dispensing pharmacies in Japan between August 2010 and July 2014 were included in the study. Based on a survey on compliance to “Furuta method,” single-blind allocation was conducted into compliance (n = 437) and non-compliance (n = 451) groups, followed by a retrospective data collection. The primary and secondary outcomes were the healing period and rates of unhealed wounds, respectively. Data was expressed as mean ± standard deviation. Two-sided log rank tests were used for between-group comparisons of PU progression, whereas Kaplan–Meier plots were used for comparison between groups. We performed rigorous adjustment for marked differences in baseline patient characteristics by propensity score (PS) matching.

**Results:**

After PS matching, patients were categorized as DESIGN-R d2 (n = 202), D3 (n = 130), D4 and 5 (n = 76), and DU (n = 76). In terms of the healing period, the patients in the compliance groups healed faster than those in the non-compliance groups in d2 (23.6 ± 36.8 vs. 32.2 ± 16.6 days; *P* < 0.001), D3 (46.8 ± 245.5 vs.137.3 ± 52.7 days; *P* < 0.001), and D4, 5 (122.5 ± 225.7 vs. 258.2 ± 292.7 days; *P* < 0.001). There were significantly lesser events of PU progression in the compliance group than in the non-compliance group (15 vs. 54; *P* = 0.003).

**Conclusions:**

“Furuta method” is the new therapeutic strategy of PU, a pharmacist intervention guidelines, may possibly increase healing rates of PUs.

## Background

Pressure ulcers (PUs) may develop when persistent pressure on bony prominences obstructs a healthy capillary flow, leading to tissue necrosis [1]. The primary cause of PUs is ischemic changes induced by pressure-related external forces, including shear force [2], and it is a common complication of immobility among the elderly [3]. Moreover, the elderly are at a high risk of developing PU because of immobility [4, 5], poor nutritional status [6, 7], and decreased body weight [5]. Despite recent research developments in the process of wound healing, treatment strategies for pressure ulcers remain inconclusive [8]. PU is a major contributor to an impaired quality of life by increasing mortality and the length of stay, burdening healthcare systems [9, 10].

An important factor in treatment resistance is the undermining formation, commonly observed in deep PUs [11]. Treatment-resistant PUs may be associated with changes in physical wound properties because of aging, such as mobility and deformity [12], and the high incidence of the undermining formation in PUs over the sacrum, coccyx, and greater trochanter [13]. Evaluation of wound deformity and mobility has been shown to improve healing following topical ointment therapy [14]; moreover, an active control of topical ointment application has been demonstrated to be important for moist wound healing in PUs [15].

In Japan, PUs have been treated by a team comprising physicians, pharmacists, and nurses, based on physical properties of the wound and using topical ointment for moist wound healing. Thus, this concept has been shown to be therapeutically effective at an improved medical cost [16]. Further, it has been spreading to pharmacists in Japan. However, there have been no detailed descriptions of interventions by pharmacists for PU treatment. Based on this background, we newly proposed the concept of topical ointment therapy by pharmacists, called “Furuta method” (Appendix), for PU, considering the physical properties and moist environment of wounds, and we promote widespread training sessions according to Furuta method in Japan. The aim of this study was to investigate the effectiveness of PU therapeutic effects treated by trained pharmacist according to “Furuta method” in Japan and to compare the results based on the compliance surveys of this “Furuta method.”

## Methods

### Setting

The study was based on compliance surveys and retrospective data collection from 37 hospitals and five dispensing pharmacies in Japan. Hospitals and dispensing pharmacies were appealed to participate via the “decunet” mailing list used by pharmacists involved in PU. Pharmacists who participated in this study underwent training sessions according to “Furuta method” in Japan. We set the suitable standards for the PU team, which comprised physicians, pharmacists, nurses, and other medical staff. Data of the National Center for Geriatrics and Gerontology (NCGG) were excluded because Furuta was involved in PU treatment. Finally, 35 hospitals and four pharmacies were included. All researchers collected data in a severely managed the personal information. The study protocol was approved by the ethics committee of NCGG.

### Compliance survey

For compliance confirmation of pharmacist intervention guidelines on PU topical ointment therapy called “Furuta methods” (see Appendix), a self-administered 10-item questionnaire including the questions was provided: 1. are you treating PU more than once a week?; 2. do you assess the wound using descriptive ulcer dermatology?; 3. do you assess ointment base properties?; 4. do you assess wound surface moisture and use the Expert Furuta Blend ointment?; 5. do you assess the physical properties of the wound?; 6. do you treat the wound using wound fixation?; 7. do you assess the wound surface and edge?; 8. do you control the moisture of the wound by assessing granulation tissue deformation by an external force?; 9. do you assess external forces applied to the wound and treat by wound fixation?; and 10. do you assess residual tissue, such as the dermis, subcutaneous tissue, and fascia? This questionnaire was completed by responding yes (1 point) or no (0 point). This questionnaire was a single-blind method, which allocated patients to the compliance (≥8 points) and non-compliance (<8 points) groups. This was followed by a retrospective data collection.

### Patients

**Fig. 1:**
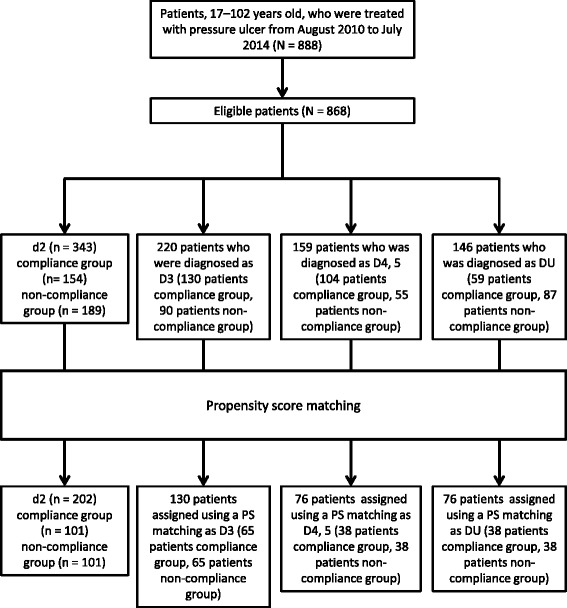
Flow chart for study group inclusion. Patients were grouped according to the DESIGN-R category of depth and were divided into two groups based on compliance survey results. Finally, patients were allocated by propensity score matching

A total of 888 consecutive patients who underwent PU treatment in Japan between August 2010 and July 2014 were enrolled in this study (Fig. 1). Only patients who met the following criteria were included: (a) patients whose diagnosis were assigned as DESIGN-R [17] ≥ d2; (b) patients who received intervention from the PU team for ≥7 days during the observational period. Patient with missing information on the following data were excluded from this study: age, sex, hemoglobin, serum albumin, site of PU, DESIGN-R score, and observation period. Thus, a total of 868 patients were included; the mean patient age was 80.0 ± 11.3 years (standard deviation, Table 1).

**Table 1:** Baseline characteristics of patients with pressure ulcers based on the DESIGN-R category of depth

	d2				D3				D4, 5				DU			
	Compliance group (n = 154)	Non-compliance group (n = 189)	Absolute standardized difference, %	*P* value	Compliance group (n = 130)	Non-compliance group (n = 90)	Absolute standardized difference, %	*P* value	Compliance group (n = 104)	Non-compliance group (n = 55)	Absolute standardized difference, %	*P* value	Compliance group (n = 59)	Non-compliance group (n = 87)	Absolute standardized difference, %	*P* value
Age (years)	82.4 ± 9.0	79.3 ± 11.6	29.9	0.06	81.1 ± 10.3	78.8 ± 12.9	19.7	0.137	80.5 ± 11.5	80.5 ± 10.8	0	0.988	79.2 ± 10.0	76.7 ± 14.7	19.9	0.265
Sex (male) (%)	51.9	54.5	3.8	0.403	56.0	55.6	0.6	0.949	40.3	36.3	5.1	0.624	45.7	56.3	15.1	0.213
Hemoglobin (g/dL)	10.9 ± 2.2	10.8 ± 2.3	4.4	0.529	10.5 ± 2.1	10.7 ± 1.9	10.0	0.57	9.9 ± 1.8	10.1 ± 1.7	11.4	0.606	9.9 ± 1.9	10.5 ± 2.1	29.9	0.107
Albumin (g/dL)	2.9 ± 0.6	2.9 ± 0.8	0.0	0.808	2.6 ± 0.6	2.6 ± 0.6	0	0.38	2.6 ± 0.6	2.5 ± 0.7	15.3	0.727	2.6 ± 0.5	2.7 ± 0.6	18.1	0.25
DESIGN-R score at baseline	9.4 ± 4.9	7.5 ± 4.8	39.1	<0.001	15.4 ± 7.3	15.4 ± 7.0	0	0.969	28.0 ± 12.5	23.0 ± 7.3	48.9	0.007	24.6 ± 10.3	20.3 ± 10.3	41.7	0.014
Observational period (day)	23.1 ± 20.1	26.1 ± 23.8	13.6	0.211	43.2 ± 42.1	57.3 ± 93.1	19.5	0.132	70.5 ± 55.6	68.8 ± 65.5	2.7	0.86	52.5 ± 59.7	57.3 ± 88.8	6.3	0.718
Locations				-				-				-				-
Sacrum	55	87	-	-	43	38	-	-	56	28	-	-	16	32	-	-
Coccyx	22	20	-	-	7	9	-	-	3	2	-	-	2	3	-	-
Greater trochanter	17	13	-	-	20	8	-	-	8	4	-	-	7	13	-	-
Heel	10	17	-	-	24	10	-	-	11	4	-	-	14	20	-	-
Ilium	4	7	-	-	5	4	-	-	4	5	-	-	5	3	-	-
Others	46	45	-	-	31	21	-	-	22	12	-	-	15	16	-	-

### Propensity score matching

To reduce treatment-selection bias and potential confounding variables, we performed rigorous adjustment for marked differences in baseline patient characteristics with propensity score (PS) matching using the following algorithm: 1:1 optimal match with a ±0.03 caliper and no replacement. Possible confounders were selected based on clinical knowledge for their potential association with the outcome of interest. The PS model was estimated using a logistic regression model that adjusted for patient characteristics, such as age, sex, hemoglobin, albumin, DESIGN-R score at baseline, and observation period. To measure covariate balance, an absolute standardized difference above 10 % represented meaningful imbalance [18, 19].

### Study variables and statistical analysis

The main outcome was a comparison of healing days, which was calculated based on a previous study [20] that used the following equation:

$$ \mathrm{Treatment}\ \mathrm{period} = \mathrm{DESIGN}\hbox{-} \mathrm{R}\ \mathrm{score}\ \mathrm{at}\ \mathrm{baseline}/\mathrm{Healing}\ \mathrm{rate} $$

$$ \mathrm{Healing}\ \mathrm{rate} = \left(\mathrm{DESIGN}\hbox{-} \mathrm{R}\ \mathrm{score}\ \mathrm{at}\ \mathrm{baseline}\right)\ \hbox{-}\ \left(\mathrm{DESIGN}\hbox{-} \mathrm{R}\ \mathrm{score}\ \mathrm{at}\ \mathrm{endpoint}\right)/\ \left(\mathrm{Intervened}\ \mathrm{period}\right) $$

DESIGN-R was used for monitoring of PU healing progression and is defined by the following components: depth, exudate, size, inflammation/infection, granulation tissue, necrotic tissue, and pocket [17]. The secondary endpoint was rates of unhealed wound.

Data were presented as mean ± standard deviation. Paired comparisons were performed using paired *t*-test for categorical variables and Mann–Whitney *U* test for continuous variables. Kaplan–Meier plots were created for evaluating the study endpoints, and the respective 95 % CI values were calculated. For the Kaplan–Meier plot for PU progression, all patients were included except for healed cases. Moreover, two-sided log rank tests were used for between-group comparisons of PU progression. All analyses were performed using SPSS version 22.0 software (SPSS Inc, Chicago, IL, USA). A two-tailed P value of <0.05 was considered statistically significant.

## Results

The mean compliance survey point was 7.3 ± 2.8, and results were collected from 19 (437 patients) and 20 (451 patients) facilities. In the questions 2, the compliance rate based on descriptive ulcer dermatology was the lowest.

Based on the DESIGN-R criteria, 343 patients were diagnosed as d2, 220 as D3, 159 as D4, 5, and 146 patients as DU (Table 1). Individuals who did and did not undergo treatment based on “Furuta method” differed for all measured characteristics.

**Fig. 2:**
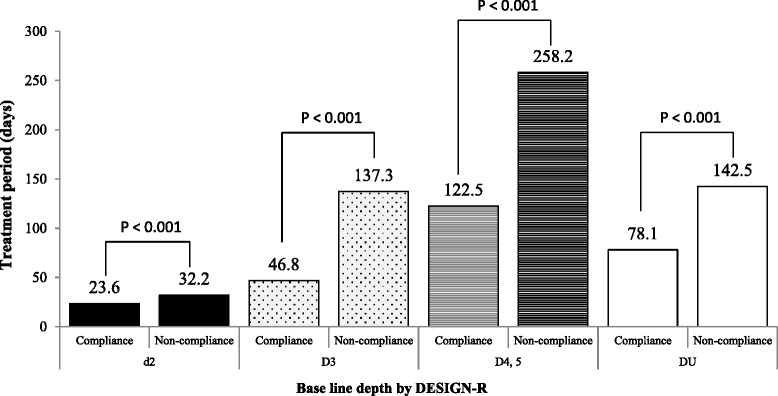
Treatment period for pressure ulcers according to DESIGN-R category of depth. The two groups in each DESIGN-R score was compared using Mann–Whitney *U* test

Table 2 shows the detailed characteristics of patients included in the final analysis: d2 (n = 202); D3 (n = 130); D4, 5 (n = 76); and DU (n = 76). The covariate balance in the matched cohort considerably improved. In Fig. 2, for each DESIGN-R group of patients, the respective duration of healing was significantly shorter in the compliance group than in the non-compliance group: d2 (23.6 ± 36.8 vs. 32.2 ± 16.6 days; *P* < 0.001); D3 (46.8 ± 245.5 vs. 137.3 ± 52.7 days; *P* < 0.001); D4, 5 (122.5 ± 225.7 vs. 258.2 ± 92.7 days; *P* < 0.001); and DU (78.1 ± 298.9 vs. 142.5 ± 79.4 days; *P* < 0.001). Moreover, wound healing was significantly different among the DESIGN-R groups (P < 0.001, data not shown).

**Table 2:** Characteristics of patients with pressure ulcers after propensity score matching

	d2			D3			D4, 5			DU		
	Compliance group (n = 101)	Non-compliance group (n = 101)	Absolute standardized difference, %	Compliance group (n = 65)	Non-compliance group (n = 65)	Absolute standardized difference, %	Compliance group (n = 38)	Non-compliance group (n = 38)	Absolute standardized difference, %	Compliance group (n = 38)	Non-compliance group (n = 38)	Absolute standardized difference, %
Age (years)	81.9 ± 8.5	82.4 ± 8.2	5.9	82.4 ± 8.6	81.8 ± 10.8	6.1	80.8 ± 12.0	80.3 ± 11.9	4.2	78.2 ± 11.0	77.6 ± 14.3	4.7
Sex (male) (%)	53.4	55.4	4.4	52.3	49.2	4.4	31.6	39.5	9.8	47.4	47.4	0
Hemoglobin (g/dL)	10.7 ± 2.1	10.9 ± 2.0	9.8	10.3 ± 1.9	10.4 ± 1.7	5.5	10.2 ± 2.1	10.3 ± 1.5	5.5	10.1 ± 2.0	10.3 ± 1.8	5.3
Albumin (g/dL)	2.9 ± 0.6	2.9 ± 0.6	0	2.6 ± 0.5	2.6 ± 0.6	0	2.6 ± 0.7	2.6 ± 0.7	0	2.6 ± 0.6	2.6 ± 0.6	9.8
DESIGN-R score at baseline	8.6 ± 4.4	8.2 ± 4.5	8.9	14.7 ± 7.7	15.1 ± 6.5	5.6	25.9 ± 11.4	25.0 ± 9.8	8.5	21.5 ± 8.4	22.1 ± 7.0	7.8
Observational period (day)	23.9 ± 21.1	22.6 ± 21.5	6.1	46.6 ± 53.4	42.1 ± 38.5	9.6	66.3 ± 47.2	63.3 ± 45.8	7.1	49.0 ± 61.5	45.1 ± 42.3	7.4
Locations												
Sacrum	39	47		23	26	-	20	22	-	11	15	-
Coccyx	14	7	-	5	4	-	1	3	-	2	2	-
Greater trochanter	7	8	-	7	4	-	2	1	-	8	4	-
Heel	9	12	-	11	7	-	3	1	-	7	10	-
Ilium	3	6	-	3	5	-	1	2	-	2	2	-
Others	29	21	-	16	19	-	11	9	-	8	5	-

**Fig. 3:**
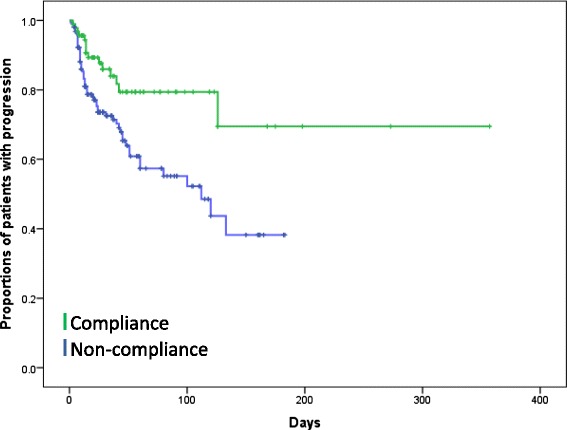
Progression of pressure ulcer. Kaplan–Meier estimates for the progression of pressure ulcer. There were 15 events of progression in the compliance group (n = 92) and 54 events of progression in the non-compliance group (n = 157; P = 0.003). *The two groups were compared by log rank test

Figure 3 shows the Kaplan–Meier plot for PU progression, according to the DESIGN-R score. The number of cases with PU progression was significantly lesser in the compliance group (n = 92) than in the non-compliance group (n =157) (15 vs. 54, respectively; *P* = 0.003; Fig. 3).

## Discussion

The results of our study demonstrate that “Furuta method,” was effective for PU treatment in terms of early complete recovery and prevention of PU progression. However, there is a need for aggressive pharmacist intervention while using this method. It is important to conduct simultaneous prevention and treatment for PU; in this study, it is noteworthy that the compliance group did not deteriorate. Pharmacists who participated in this study, result of trained workshop according to Furuta methods, more improved assessment of topical ointment therapy considering the external force for PUs were compared with similar factors in the non-compliance group.

In a previous report conducted at NCGG, treatment periods according to National Pressure Ulcer Advisory Panel (NPUAP) stage definitions were 10 ± 6 days for Stage II, 43 ± 17 days for Stage III, and 80 ± 32 days for Stage IV [20]. In our study, the mean duration of healing in the compliance group was 23.6 ± 36.8 days in patients with d2, 46.8 ± 245.5 days in patients with D3, and 122.5 ± 225.7 days in patients with D4 and D5. In a previous study, only NCGG data (based on “Furuta method”), encompassed a wide range of treatment periods that was comparable with the present study. We believe the low compliance observed with “Furuta method” may be influenced by the treatment period. Further, the compliance rate with descriptive ulcer dermatology was the lowest, and this pathological assessment was not routinely performed by general healthcare workers. It is necessary to educate pharmacists and general healthcare workers regarding these techniques. A randomized single-blind controlled trial reported 8-week healing rates for Stage II ulcers of only 50 % in both groups [21]. Moreover, Horn *et al.* reported treatment failure rates for Stage III and IV ulcers of 90 % at 8 weeks [22]. These reports mainly investigated dressing therapy for PU and did not evaluate wound assessment according to wound physical properties and descriptive ulcer dermatology. It has been proposed that topical ointment therapy according to Furuta methods had a greater efficacy than dressing therapy.

Furthermore, in this study, there were significantly fewer cases of PU progression in the compliance group than in the non-compliance group (15 vs. 54; *P* = 0.003). Thus, we believe that an appropriate wound assessment according to “Furuta method” has utility in preventative management.

This study has several limitations. First, it is a retrospective, albeit multicenter, study; hence, there is a potential for bias. This was minimized by performing PS matching, which has been previously shown to be useful [23]. Second, this study did not consider the involvement of the nutritionist and physical therapist. Therefore, prospective trials that consider these other factors are necessary in the future.

## Conclusion

We introduce a concept of PU topical ointment therapy, called “Furuta method,” which considers the physical properties and moist environment of wounds and pharmacist intervention guidelines and may be aided by pharmacist participation. This method may possibly lead to an increase in PU healing rates.

## Appendix

### Pharmacist intervention guidelines on pressure ulcer topical ointment therapy “Furuta method”

#### Wound assessment

It is desirable to examine the wound at least once a week based on descriptive ulcer dermatology [24].

1. **Wound physical property** [12]: Assess by wound mobility and deformity. Wound mobility is the movement of the wound from the bony prominence, whereas wound deformity is a change in the three-dimensional shape of the wound and can result in undermining formation, a characteristic of deep pressure ulcers. Layered granulation tissue arising from wound deformity attenuates topical ointment therapy.
2. **Wound fixation** [25]: Wound fixation is defined as alleviation of wound deformity by exogenous materials. It is classified as traction, anchor, and insertion. Wound fixation by traction is performed using an elastic bandage. Wound fixation by anchor also alleviates direct pressure on the wound using sponge (Reston™, Sumitomo 3 M, Tokyo, Japan or Prosoft™, NICHIBAN, Tokyo, Japan). Wound fixation by insertion is usually performed using materials of an appropriate hardness and absorbability, such as chitin cotton and alginate foam. For each case, the external force of the pressure ulcer was evaluated in order to select the appropriate wound fixation method.
3. **Residual tissue** [24]: To evaluate and eliminate necrosis in residual tissue, such as the dermis, subcutaneous tissue, and fascia. However, physical properties of the wound are stabilized in the dermis.
4. **Wound surface and edge**: To evaluate the several properties of the wound surface and edge, such as edematous, sclerotic, dry, glossy, or hemorrhagic granulation tissue; flat with smooth edge sunken with smooth edge or sunken with indented edge; and fibrin-coated film. Based on the evaluation of the wound surface and edge, the moisture of the wound was controlled by the characteristics of the ointment base (active or passive absorption) [26].
5. **Granulation tissue**: The granulation tissue is deformed by an external force. Edematous granulation tissue formation by an external force is necessary to consider the measures of preventing the cause of the external force. Based on the evaluation of the granulation tissue, the moisture of the wound was controlled using characteristics of the ointment base (active or passive absorption) [26], and the external force was controlled by wound fixation [25].
6. **Pocket**: A wound pocket is mainly formed by an external force. Based on the evaluation of the external force on the pocket, the moisture of the wound was controlled using characteristics of the ointment base (active or passive absorption) [26], and the external force was controlled by wound fixation [25].

#### Topical agent assessment

The selection of a topical ointment was based on the “Japanese society of pressure ulcers Guidelines for the Prevention and Management of Pressure Ulcers” [27]. As necessary, wound assessment based on descriptive ulcer dermatology [24] considered the use of Expert Furuta Blend Ointment [28]. Exudate formation in the wound was confirmed by checking the top dressing and gauze.

1. Shallow pressure ulcer
  1. **Inflammation and infection:** Povidone–iodine sugar ointment, dextranomer beads, cadexomer iodine beads, iodine–potassium iodide gel, and iodoform gauze
  2. **Necrotic tissue**
    Under the wound surface moisture: silver sulfadiazine cream, and iodoform gauze with saline
    Over the wound surface moisture: iodoform gauze and bromelain ointment
  3. **Epithelialization**
    Under the wound surface moisture: lysozyme hydrochloride ointment with sulfadiazine ointment (3:7), bucladesine sodium ointment, alprostadil alfadex ointment, trafermin splay, lysozyme hydrochloride ointment with iodine gel (1:1), and hydrocolloid dressing
    Over the wound surface moisture: povidone–iodine sugar ointment and urethane foam dressing
2. Deep pressure ulcer
  1. **Inflammation and infection**: povidone–iodine sugar ointment, dextranomer beads, cadexomer iodine beads, iodine–potassium iodide gel, and iodoform gauze
  2. **Necrotic tissue**
    Surgical debridement for black necrotic tissue
    Under the wound surface moisture: silver sulfadiazine cream and iodoform gauze with saline.
    Over the wound surface moisture: iodoform gauze, bromelain ointment, povidone–iodine sugar ointment, dextranomer beads, cadexomer iodine beads, and iodine–potassium iodide gel
3. **Formation of granulation tissue**
  Edematous granulation tissue: povidone–iodine sugar ointment, povidone–iodine sugar ointment with dextranomer beads (4:1), and tretinoin tocoferil ointment with dextranomer beads (3:2)
  Dry granulation tissue: tretinoin tocoferil ointment, trafermin spray, povidone–iodine sugar ointment with tretinoin tocoferil ointment (3:1), tretinoin tocoferil ointment with sulfadiazine ointment (3:7), and tretinoin tocoferil ointment with dextranomer beads (3:2)
4. **Epithelialization**
  Under wound surface moisture: lysozyme hydrochloride ointment with sulfadiazine ointment (3:7), bucladesine sodium ointment, alprostadil alfadex ointment, trafermin spray, lysozyme hydrochloride ointment with iodine gel (1:1), and hydrocolloid dressing
  Over the wound surface moisture: povidone–iodine sugar ointment, and urethane foam dressing
